# The Role of Host and Fungal Factors in the Commensal-to-Pathogen Transition of *Candida albicans*

**DOI:** 10.1007/s40588-023-00190-w

**Published:** 2023-03-31

**Authors:** Ilse D. Jacobsen

**Affiliations:** 1 Research Group Microbial Immunology, Leibniz Institute for Natural Product Research and Infection Biology, Jena, Germany; 2 Institute of Microbiology, Friedrich Schiller University, Jena, Germany

**Keywords:** Pathogenesis, Candidiasis, Mucosa, Oral cavity, Vulvovaginal, gut

## Abstract

**Abstract:**

**Purpose of Review:**

The fungus *Candida albicans* has evolved to live in close association with warm-blooded hosts and is found frequently on mucosal surfaces of healthy humans. As an opportunistic pathogen, *C. albicans* can also cause mucosal and disseminated infections (candidiasis). This review describes the features that differentiate the fungus in the commensal *versus* pathogenic state and the main factors underlying *C. albicans* commensal-to-pathogen transition.

**Recent Findings:**

Adhesion, invasion, and tissue damage are critical steps in the infection process. Especially invasion and damage require transcriptional and morphological changes that differentiate *C. albicans* in the pathogenic from the commensal state. While the commensal-to-pathogen transition has some conserved causes and features in the oral cavity, the female urogenital tract, and the gut, site-specific differences have been identified in recent years.

**Summary:**

This review highlights how specific factors in the different mucosal niches affect development of candidiasis. Recent evidence suggests that colonization of the gut is not only a risk factor for systemic candidiasis but might also provide beneficial effects to the host.

## Introduction

The fungus *Candida albicans* is found on mucosal surfaces of healthy humans and other warm-blooded animals. Despite its versatile metabolism and ability to utilize a wide variety of nutrient sources, it has only rarely been isolated from the environment [1]. Thus, it appears that *C. albicans* has evolved to live in close association with animal hosts. Colonization of humans usually occurs early, within the first months of life [2, 3]. The main sites of mucosal colonization are the oral cavity and the gut [4–6]; in addition, *C. albicans* is found in the vaginal cavity [7–10]. As an opportunistic pathogen, *C. albicans* can also cause infections (candidiasis). Infections can be limited to the mucosa, such as oral/oropharyngeal and vulvovaginal candidiasis, or be characterized by translocation across mucosal barriers, such as *C. albicans* peritonitis, candidemia, and disseminated (systemic) candidiasis [11]. While intestinal colonization is common and found in 40–60% of healthy individuals [4, 5], mucosal candidiasis occurs infrequently and is often associated with specific risk factors such as antibiotic treatment, impaired epithelial barrier function, or altered immune responses [12]. Candidemia and disseminated candidiasis are mainly observed in patients with severe underlying medical conditions requiring hospitalization and are rare but life-threatening events [11]. Both mucosal and systemic infections are endogenous, caused by *C. albicans* strains that colonized the host before the onset of disease rather than by newly acquired strains with specific virulence properties [13, 14••]. This raises the question why and how *C. albicans* turns from a commensal, which relies on its host as essential niche, to a pathogen. Development of candidiasis is not only associated with reduced host defense, but also with transcriptional changes of the fungus in response to a changing host environment, which facilitate activities of the fungus associated with the infection process that are largely absent during colonization [15]. This change in fungal interactions with the host is termed commensal-to-pathogen transition [15]. In this review, I will describe the main steps of *C. albicans* commensal-to-pathogen transition and discuss known factors that influence this transition at different body sites.

## Steps and Features of *C. albicans* Commensal-to-Pathogen Transition

As a commensal, *C. albicans* is present on mucosal surfaces, but not necessarily in direct contact with epithelial cells. In the gut, the extensive mucus layer effectively separates most microbes, including *C. albicans*, from epithelial cells [16, 17•]. The oral cavity, however, lacks a distinct mucus layer, and instead salivary flow physically removes microbes [18]. In this setting, adherence to epithelial cells is necessary for establishment of persistent colonization. *C. albicans* expresses several factors including Als3 [19], Hwp1 [20], and Iff4 [21, 22] that facilitate adhesion. Adhesion is not sufficient to cause tissue damage and therefore can be considered a colonization factor. However, it is also prerequisite for subsequent invasion, the first step of infection.

Invasion of epithelial cells by *C. albicans* appears to be a key step in commensal-to-pathogen transition during oral candidiasis as *C. albicans* mutants with reduced capacity to invade epithelial cells in vitro are usually attenuated in virulence in murine models of oral candidiasis [23]. Two distinct processes facilitate invasion [24]: (i) induced endocytosis, driven by the host cell following interaction of fungal Als3 and Ssa1 with host proteins such as cadherins, EGF receptor, and HER2 (human epidermal growth factor receptor 2) [25–27], and (ii) active penetration by the fungus, which requires the formation of hyphae that can directly penetrate epithelial cells or grow between intercellular junctions [28, 29]. Hypha formation not only facilitates active penetration, but also increases adhesion by increased expression of adhesins [30, 31]. Furthermore, filamentation is also critical for damage of epithelial cells following invasion. Damage can be mediated by mechanical disruption of host cell integrity by piercing hyphae [24], activation of specific cell-death pathways [32], and the expression of hypha-specific virulence factors such as the peptide toxin candidalysin [33••, 34]. Invading *C. albicans* hyphae accompanied by destruction of the superficial epithelium are typical for oral candidiasis [35] and vulvovaginal candidiasis [36•, 37] and also observed in internal organs during systemic candidiasis [38]. Given the prominent role of filamentation in invasion, damage, and clinical disease, hypha formation can be considered as a key step in the transition of *C. albicans* from a commensal stage to infection [38].

Filamentation, invasion, and tissue damage elicit an inflammatory host response that is essential for fungal clearance, yet also contributes to the development of clinical symptoms [12, 39]. The underlying mechanisms have been studied in detail in the context of oral infections: Oral epithelial cells detect *C. albicans* yeast cells and respond by intracellular signaling, but only viable hyphae induce activation of the transcription factor c-Fos and the MAPK phosphatase MKP1, which results in the release of pro-inflammatory cytokines [40, 41], and recruitment of professional immune cells [42]. In addition, fungal burden needs to reach a threshold level to achieve full activation [41]. In healthy individuals, the abundance of *C. albicans* on mucosal surfaces is usually low, whereas candidiasis is commonly associated with increased fungal burden [14, 43, 44]. A functional relationship between fungal burden and candidiasis is supported by the observation that antibiotic treatment, which is commonly associated by expansion of commensal fungi due to reduced bacterial competition, is a risk factor for candidiasis [11, 43, 44]. While increased fungal load might increase the likelihood of infection events stochastically, the differential response of epithelial cells to the fungal load suggests more specific mechanisms linking fungal load and candidiasis. If that is the case, increased fungal proliferation could be considered a key step in the commensal-to-pathogen transition of *C. albicans.*

## *C. albicans* Commensal-to-Pathogen Transition in the Oral Cavity

**Fig. 1:**
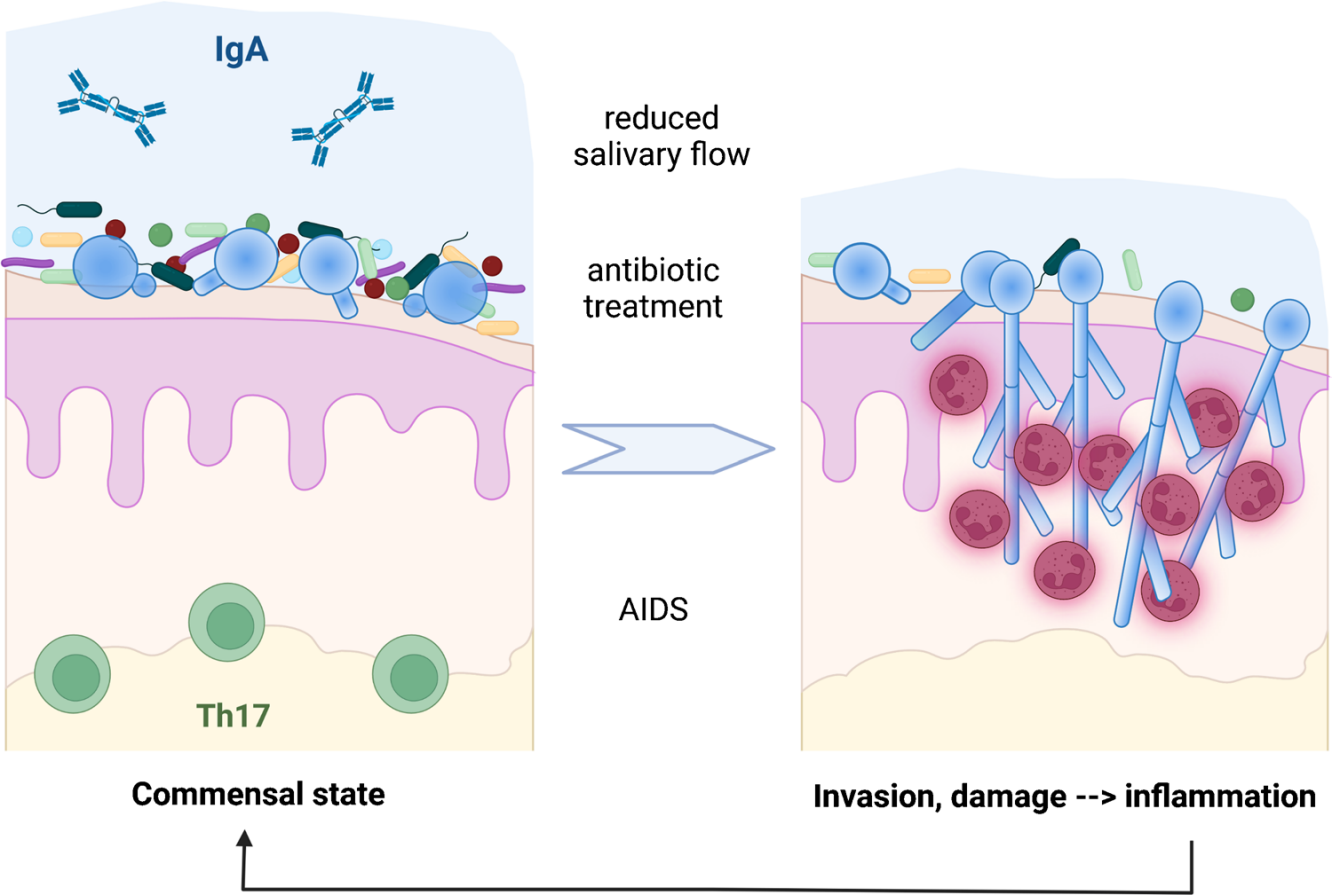
Host-pathogen transition in the oral cavity. In the commensal state, commensal bacteria, secretory IgA, and surveillance by IL-17-producing T cells result in low fungal burden and absence of invasion-driven tissue damage (left). Reduced IgA (for example due to reduced salivary flow), increased fungal burden due to antibiotic treatment, and T helper cell deficiency (AIDS) are associated with increased fungal burden, filamentation, and tissue damage (right). Subsequent neutrophil recruitment leads to inflammation, followed by reduction of fungal burden and restoration of the commensal state. Created with BioRender.com

*C. albicans* is generally considered to be the most frequent fungal colonizer of the oral cavity [3, 6], but the prevalence in healthy individuals varies from around 20% in an adult cohort in Hong Kong [45] to 40 to 60% in European adults [46, 47] reported in culture-based studies. In specific groups like denture wearers, patients undergoing radiotherapy for head or neck cancer, and HIV-positive individuals, *C. albicans* is isolated more frequently [48–50]. These differences could be due to colonization occurring in association with the underlying conditions but could also reflect increased fungal burden resulting in more frequent detection. The latter is supported by the negative correlation of salivary flow and fungal abundance in patients after radiotherapy [51] and the higher abundance of *C. albicans* in HIV-positive patients compared to HIV-negative controls [50, 52]. However, these conditions are not only associated with increased *C. albicans* prevalence and fungal burden, but also represent risk factors for oral candidiasis due to impaired immune responses [44]. Indeed, high fungal burden alone does not necessarily indicate a shift from commensalism to infection: *C. albicans* strains differ in their ability to colonize the murine oral cavity, with some strains achieving persistent colonization at comparatively high fungal burden (commensalism), while others are cleared [53••]. Persistent colonization coincides with the absence of tissue damage and inflammation [53••]. Interestingly, passaging of a highly invasive *C. albicans* strain that is cleared from the oral mucosa in immunocompetent mice results in genomic changes associated with reduced filamentation [54]. Similarly, genetic modification leading to reduced filamentation facilitated enhanced colonization, and conversely, de-repression of the hyphal program in a commensal strain resulted in increased virulence and faster fungal clearance [55•]. Thus, morphological transition from yeast to hypha is a central step in the commensal-to-pathogen transition of *C. albicans* in the oral cavity. This is linked to the damage-inducing capacity of hyphae, especially the production of candidalysin, which induces upregulation of inflammatory cytokine production by oral epithelial cells [56••]. The resulting inflammatory immune response might contribute to clinical symptoms during the acute stage of infection, but in the long term selects against strains that undergo yeast-to-hypha transition (Fig. 1).

While fungal clearance during acute oropharyngeal candidiasis relies on innate immune components such as neutrophils and antimicrobial peptides [57–59], maintenance of homeostasis relies on adaptive immune responses to commensal *C. albicans* [60]. Long-lived IL-17-producing T cells are induced in response to colonization [61, 62] and facilitate immunosurveillance of *C. albicans* as a commensal without inducing inflammation [60] (Fig. 1). It seems plausible that these cells could also initiate fast responses to localized invasion events, and thereby prevent clinical infection; however, if localized invasion occurs, healthy hosts are unknown. Such events might be prevented by B cells. IgA+ cells have been shown to migrate to foci of fungal colonization in the oral cavity of mice, and oral colonization of mice leads to increased levels of cross-specific IgA in the saliva and the tongue [63•]. In vitro, binding of IgA to *C. albicans* prevented fungal epithelial adhesion and invasion and dampened the pro-inflammatory epithelial response [63•]. Furthermore, B cell deficiency or depletion in mice results in higher oral fungal burden [63•]. Thus, B cell responses might contribute to the maintenance of commensalism by both preventing the initial steps of the commensal-to-pathogen shift and by controlling the fungal load (Fig. 1).

Disturbance of the oral bacterial community by the prolonged use of broad-spectrum antibiotics is an important risk factor for oral candidiasis [64]. In the past, this has been mainly linked to the reduction of antagonistic bacteria, which limit fungal growth and also directly affect virulence features such as filamentation (recently reviewed in [65]). More recently, microbiome studies have revealed that *C. albicans* colonization also affects bacterial communities in the oral cavity [66••, 67••]. *C. albicans* colonization supports colonization with Streptococcus oralis in mice [68] and leads to expansion of endogenous Enterococcus faecalis [66••]; in both cases, the presence of the bacteria led to augmented severity of oral candidiasis. This underlines the importance of the mucosal microbiome not only as part of the host defense system, but also as an environmental factor to which *C. albicans* responds and which can influence the transition from commensalism to infection.

## Special Aspects of Vulvovaginal Candidiasis

Vulvovaginal candidiasis (VVC) is characterized by acute inflammation leading to disease symptoms such as vaginal itching, burning, pain, redness, and vaginal discharge [43]. VVC is common and estimated to afflict 70–75% of women at least once during their lives, with over 5% experiencing several episodes within a year (recurrent vulvovaginal candidiasis, RVCC) [43, 69, 70]. Most cases occur in otherwise healthy, immunocompetent women [43, 69, 70], which differentiates VVC from oropharyngeal candidiasis. Predisposing factors include the hormonal status (especially use of estrogen as hormone replacement therapy or oral contraceptive), and use of antibiotics [43], but a large proportion of cases occurs without a known underlying cause [70].

Similar to oral epithelial cells, the infection of vaginal epithelial cells with *C. albicans* is characterized by adhesion, invasion, and damage [71], and the response of vaginal epithelial cells to *C. albicans* in vitro depends on both hypha formation and fungal burden [72]. Yeast-to-hypha transition is also essential for inflammatory responses in murine models of VVC [73], and increased filamentation is observed in women with VVC compared to asymptomatic controls [36•, 37]. Inflammation furthermore depends on proteases and the toxin candidalysin, which not only mediates damage but also directly induces inflammasome-dependent responses of vaginal epithelial cells [74, 75•]. This largely resembles the interaction of *C. albicans* with oral epithelial cells, but some differences exist: Vaginal epithelial cells respond to the same fungal burden with lower cytokine production than oral cells [72]. The production of alarmins and pro-inflammatory cytokines by the vaginal epithelial cells induces neutrophil migration, which is at least partly responsible for development of clinical symptoms [76–78]. Furthermore, the role of Il-17 signaling is less clear than in oral infections (recently reviewed in [79]), and recruited neutrophils fail to reduce fungal burden (recently reviewed in [78••, 79]). In vitro and in vivo studies conducted within the last two decades, including a challenge study in human volunteers [80], suggest a high tolerance of the vaginal epithelium to *C. albicans*, with VVC/RVVC requiring three components: (i) high fungal burden, (ii) invasion, and (iii) an aggressive innate immune response [78••, 79, 81, 82].

**Fig. 2:**
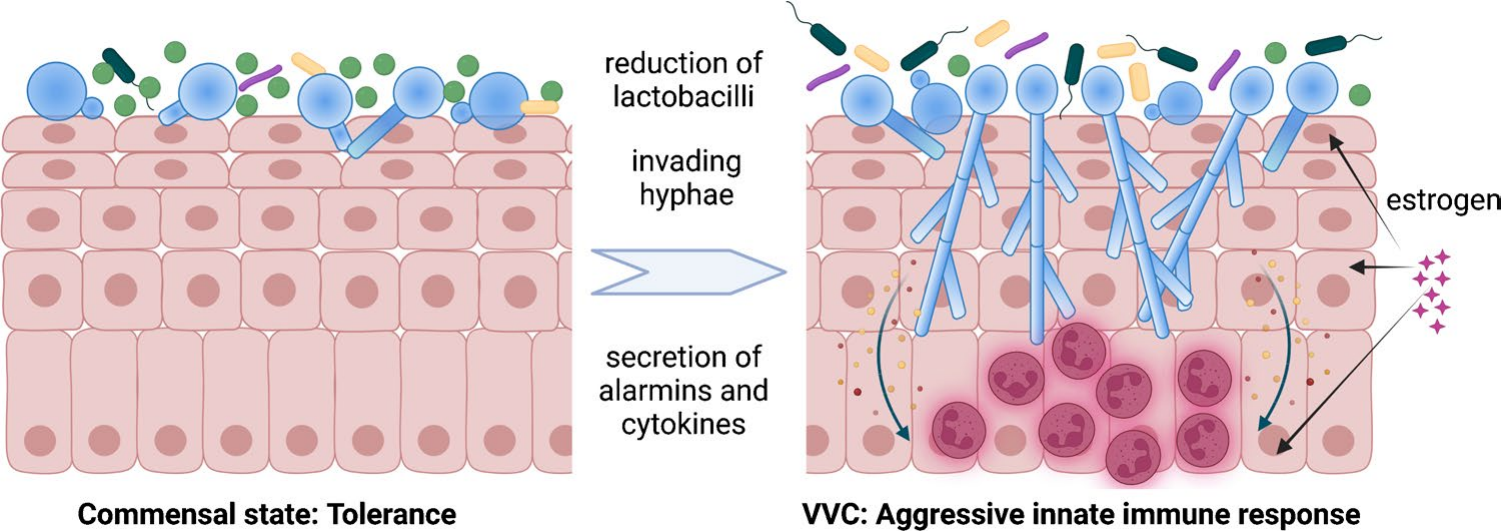
Development of vulvovaginal candidiasis. In the commensal state, vaginal epithelial cells tolerate *C. albicans* in the absence of fungal filamentation (left). Reduced numbers of lactobacilli (green) and high estrogen are associated with high fungal burden, filamentation, and tissue damage. Production of alarmins and cytokines by epithelial cells leads to neutrophil recruitment and inflammation-driven clinical symptoms (right). Created with BioRender.com

The vaginal microbiota is distinct from other body sites and in many women dominated by lactobacilli [83] (Fig. 2). *Lactobacillus* spp. produces lactic and other organic acids that contribute to the low pH within the vaginal cavity [84]. Low pH (< 4.5) inhibits *C. albicans* yeast-to-hypha transition [85] and thereby impacts adhesion and invasion. In addition to organic acids, lactobacilli can produce bacteriocins and hydrogen peroxide, which all contribute to a direct antifungal effect of lactobacilli (reviewed in [86, 87]). Furthermore, by competing for adhesion sites, lactobacilli were reported to reduce adhesion of *C. albicans* to epithelial surfaces [88]. Several *Lactobacillus* spp. occur in the female reproductive tract, and substantial inter- and intraspecies diversity with functional consequences has been described [89]. By disturbing the vaginal microbiota composition and reduction of lactobacilli, antibiotics could promote an increase in fungal burden and filamentation, thereby explaining the clinical link between the use of antibiotics and VVC. Consistent with this, several studies found significant differences in *Lactobacillus* spp. colonization levels or changes in species distribution between women with or without VVC (reviewed in [79]).

Besides antibiotics, high estrogen levels are a risk factor for VVC, and establishment of VVC in rodent models is estrogen-dependent. Estrogen exerts multiple effects on *C. albicans* itself, immune cells, and the vaginal environment (reviewed in [90]); it might therefore affect fungal activities leading to pathogenesis, the environment *C. albicans* responds to, and the immune response [79]. Changes in the immune response, with a shift from tolerance to inflammation, could underly episodes of VVC without clinical risk factor; genetic components have been identified for RVVC [91, 92•] and might underly the higher prevalence of VVC in African American compared to Caucasian women in the USA [93, 94].

## Colonization of the Gut: a Source for Disseminated Infection

**Fig. 3:**
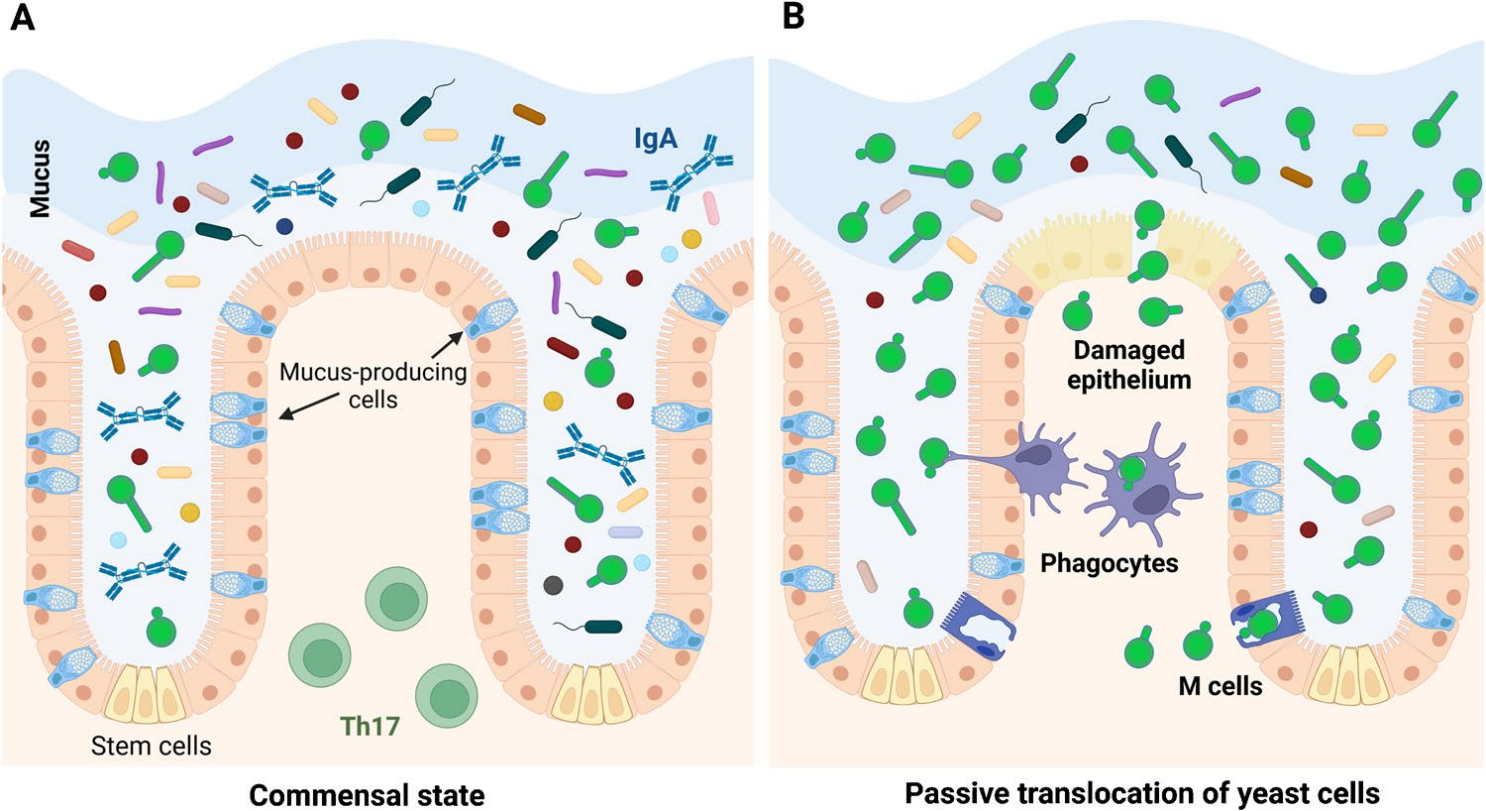
*Candida albicans* in the gut. **A** In the commensal state, the fungus is found in numbers as yeast and short hyphae. Mucins and secretory IgA favor the yeast form. **B** Reduction of the bacterial microbiota facilitates *C. albicans* expansion. In the absence of active penetration, yeast cells might translocate through damaged epithelium, or *via* uptake by M cells or phagocytes. Created with BioRender.com

Several studies provide evidence that the human gastrointestinal (GI) tract serves as a source for systemic candidiasis [13, 14••, 95]. This concept is supported by studies using mice, which demonstrate that *C. albicans* can translocate from the murine GI tract and establish systemic infection in severely immunocompromised animals [96–98]. In vitro interaction of *C. albicans* with enterocytes is similar to the interaction with other types of epithelial cells, with adhesion being followed by hypha formation, invasion, and damage driven by candidalysin [99•]. Contrary to oral epithelial cells, tight junctions prevent fungal contact to cadherins on the enterocyte surface, thereby preventing uptake by induced endocytosis [100], resulting in active penetration driven by hyphae as the main route of invasion [101, 102]. This facilitates translocation across enterocyte layers in vitro [99•]. In murine models, however, only one study reported large-scale invasion of the intestinal mucosa [103]. Invading *C. albicans* hyphae were rarely observed in intestinal tissue by others [17, 104, 105•, 106••, 107], although *C. albicans* occurs as a mixture of yeast and up to 60% hyphae [105•] (Fig. 3A). This questions whether the classical commensal-to-pathogen transition occurring in vitro is essential for translocation in vivo. Alternative routes of translocation that have been discussed are passive entry via microfold cells or phagocytes [108, 109], as well as significant impairment of the epithelial barrier function due to underlying disease or mechanical lesions [98, 108] (Fig. 3B). These mechanisms would not require filamentation-driven active invasion and would allow translocation of yeast cells. As yeast cells are thought to be more efficiently transported via the blood stream [38, 110], the substantial proportion of yeast cells in the GI tract in combination with passive translocation could explain why the gut, but not mucosal infection of the oral or vaginal cavity, seems to serve as a source for systemic infection: Mucosal infection is characterized by hypha formation; for efficient dissemination, these hyphae would have to revert to the yeast form upon invasion of blood vessels despite the presence of serum as a strong inducer of filamentation [111]. Passive translocation of yeast cells across the intestinal epithelial barrier would not require hypha-to-yeast transition and might therefore be more effective in delivering yeast cells to the blood stream. It is also possible that localized invasion events accompanied by hypha-driven tissue damage facilitate the passive translocation of neighboring yeast cells [108].

Depletion of bacteria by antibiotics is required to achieve high levels of *C. albicans* intestinal colonization in laboratory mice [112], and changes in microbiota composition or activity likely mediate the effect of specific diets on fungal burden [113]. This reflects the situation in humans, where expansion of *Candida* in the GI tract was shown to precede translocation into the bloodstream [14••]. Gut bacteria, such as some strains of *Bacteroides thetaiotaomicron* and lactobacilli, have been shown to reduce *C. albicans* colonization of the gut (recently reviewed in [114]). Based on what is known about lactobacilli in the context of VVC (see above), *Lactobacillus* spp. could additionally affect expression of *C. albicans* virulence factors and thereby promote commensalism. However, colonization of germ-free mice results in high fungal burden but no mucosal pathology, and the yeast morphology dominates in the GI tract of these animals [17•]. The group of K. Ribbeck showed that mucus, specifically mucin O-glycans, suppresses adhesion and filamentation [115••, 116], thereby identifying an abundant host factor in the GI tract that directly represses commensal-to-pathogen transition. Hyphae are also the main target of secreted IgA produced in response to *C. albicans* intestinal colonization, and the competitive fitness of *C. albicans* negatively correlates with the filamentation potential [106••, 113, 117••, 118••]. In addition to antibody production, GI tract colonization with *C. albicans* induces robust Th17 responses, which reduce susceptibility to systemic candidiasis and disseminated infection with extracellular bacteria (reviewed in [119]) (Fig. 3A). While these responses can aggravate inflammatory conditions such as asthma or colitis, the beneficial effects of colonization-induced immunological changes might dominate for most individuals [119]. Interestingly, priming of systemic Th17 immunity by *C. albicans* colonization requires dynamic fluctuation of the expression of *UME6*, which is a transcriptional regulator of filamentation, and exposure of the immunogenic cell wall moieties mannan and β-glucan [120••]. The mixture of yeast and hyphae observed in the GI tract might therefore reflect morphological plasticity linked to fungal virulence in other niches but necessary for the beneficial effects of commensal colonization. Taken together, this suggests that *C. albicans* usually behaves as a commensal within the GI tract and that specific host responses select for commensal behavior characterized by the absence of overt filamentation.

## Conclusion

The behavior of *C. albicans* on mucosal surfaces is shaped by the interaction with the host tissue, the local microbiota, and fungal factors. On the host side, the response of the epithelia to the presence of the fungus can range from a tolerogenic state, typically associated with fungal commensalism and low fungal burden, to the release of inflammatory signals in response to high fungal burden, invasion, and subsequent tissue damage. Fungal commensalism is sufficient to induce adaptive immune responses; antibody responses are mainly directed against the invasive, damaging hyphal form, thereby contributing to selection of non-damaging fungal behavior. In parallel, Th17 responses prepare the host for possible invasion events by facilitating enhanced recruitment and activation of neutrophils as antifungal effector cells. In principle, this cross-talk between epithelial, adaptive, and innate immune cells allows the host to efficiently respond to fungal-driven tissue damage while tolerating non-threatening commensal behavior of *C. albicans*. However, a propensity of the epithelium to produce inflammatory signals can shift the balance towards an inflammatory state; this appears to be a main factor in the development of VVC and RVVC, in which overt inflammation and neutrophil recruitment without control of fungal burden contribute to clinical disease. Identification of factors or mechanisms that drive or augment the inflammatory response in VVC, and especially RVVC, could provide novel targets for treating this type of mucosal candidiasis. It is furthermore intriguing that the intestinal environment seems to differ from both the oral and the vaginal cavity in so far, as that high fungal burden and the presence of hyphae are tolerated by the host immune system. This is likely linked to the lack of extensive mucosal invasion, which seems to be independent of bacterial microbiota, and possibly an effect of adaptation to this specific niche.

Technological advancements facilitating microbiome research allowed to study *C. albicans* in the context of the microbiota. The main focus of such studies has been the identification of microbes that limit fungal burden, or inhibit fungal activities involved in the commensal-to-pathogen transition, and thereby promote commensal behavior. Changes in microbiota composition explain the clear link between antibiotic treatment and candidiasis in the clinical setting and provide a rationale for investigating the therapeutic efficacy of probiotic bacteria. More recently, synergistic interactions between *C. albicans* and facultatively pathogenic bacteria have been identified—this suggests that the view that bacteria is “good” because they control fungal growth and might be over-simplified. It is possible that specific fungal-bacterial combinations are associated with an increased risk of infection, and identification of such combinations would allow to identify patients at risk.

While probably not yet comprehensive, a large number of fungal factors have been identified that contribute to adhesion, invasion, and damage and are thereby involved in the commensal-to-pathogen transition. However, most studies were based on *C. albicans* SC5314, a strain characterized by strong filamentation, and high invasion rates and damage capacity. Recent studies using additional clinical isolates or investigating genetic alterations in SC5314 following prolonged mucosal colonization support the concept that pathogenic behavior negatively impacts colonization levels and that less invasive strains are more efficient colonizers. This raises the question why *C. albicans* maintained the ability to (i) undergo yeast-to-hyphae transition and (ii) produce damage-inducing factors like candidalysin, although this triggers inflammatory responses that lead to reduction of fungal burden. It is possible that filamentation and other virulence factors are either necessary for the competition with bacteria or other fungi or that limited expression and the subsequent host response shape a specific niche for *C. albicans* in the GI tract.

In summary, despite significant progress in our understanding of *C. albicans* as a commensal, and of the factors that promote or allow the shift from commensal to pathogen, many important questions remain.
